# Supplementary material for: Bagasse Cellulose Composite Superabsorbent Material with Double-Crosslinking Network Using Chemical Modified Nano-CaCO3 Reinforcing Strategy
Source: Nanomaterials (Basel). 2022 Apr 25;12(9):1459. doi: 10.3390/nano12091459 (PMC9104651; doi:10.3390/nano12091459)
Supplement: Supplementary file 1 [file nanomaterials-12-01459-s001.zip › nanomaterials-1686266-supplementary.pdf]

Supplementary material

Bagasse cellulose composite superabsorbent material double-crosslinking network using chemical modified nano-$CaCO_3$ reinforcing strategy

Xinling Xie [1,*], Li Ma [1], Yongmei Chen [2], Xuan Luo [1], Minggui Long [2], Hongbing Ji [1,3] and Jianhua Chen [4]

[1] School of Chemistry and Chemical Engineering, Guangxi Key Laboratory of Petrochemical Resource Processing and Process Intensification Technology, Guangxi University, Nanning 530004, China; mali15956442057@163.com (L.M.); luoxuan@gxu.edu.cn (X.L.); jihb@mail.sysu.edu.cn (H.J.)

[2] Guilin Zhuorui Food Ingredients Co., Ltd., Guilin 541001, China; lilychym-123@163.com (Y.C.); longmg@126.com (M.L.)

[3] Fine Chemical Industry Research Institute, School of Chemistry, Sun Yat-sen University, Guangzhou 510275, China

[4] School of Resources, Environment, and Materials, Guangxi University, Nanning 530004, China; jhchen@gxu.edu.cn

* Correspondence: Correspondence: xiexinling@126.com

† These authors contributed equally to this work.

1. The cyclic performance test method for CAAMC

The dried and weighed sample (0.01 g) was soaked in 250 mL of deionized water until it reached swelling equilibrium. After it reached swelling equilibrium, the resin was weighed, W_i (g). The above process was repeated 4 to 5 times, and the ratio of each water absorption capacity to the first water absorption capacity was calculated. Three measurements were repeated for each sample. The calculation formula is shown in Eq. S1:

$$\text{repetitive}(\%)=\frac{w_i-w_d}{w_0-w_d}\times 100\% \quad \text{(S1)}$$

where W_d(g) is the mass of the dried sample and W_0(g) is the weight after the first absorption of water.

2. The test method for water retention of CAAMC at different temperatures

The following method was used to test the water retention of the superabsorbent resin. The dried sample (0.01 g) was immersed in excess deionized water, and we waited until the resin reached swelling equilibrium at room temperature. Then, we filtered out the excess water with a sieve. The resin W_0(g) with swelling equilibrium was weighed and placed in a petri dish, and then the petri dish with the superabsorbent resin was heated at 20, 30, 40, 50, and 60 °C. The resin was weighed at regular intervals (W_t) using Eq. S2 to calculate the water retention at different temperatures:

$$\text{water retention}(\%)=\frac{w_t-w_d}{w_0-w_d}\times 100\% \quad \text{(S2)}$$

where W_t is the mass of the superabsorbent resin at time t, g, and W_d is the mass of the dry superabsorbent resin, g.

3. Swelling Kinetic Fitting

The kinetic experiment data were analyzed by the pseudo-first-order (Eq. (S3)) and pseudo-second-order kinetic models (Eq. (S4)), as below:

$$Q_t = Q_e(1-e^{k_1 t}) \quad \text{(S3)}$$

$$\frac{t}{Q_t}=\frac{1}{k_2 Q_e^2}+\frac{1}{Q_e}t \quad \text{(S4)}$$

where Q_e (g/g) and Q_t (g/g) are the water absorbency of the prepared CAAMC at equilibrium and at time t (min), respectively. k_1(min^{-1}) and k_2 (mg/g min^{-1}) are the rate constants of the pseudo-first order and pseudo-second-order kinetic models, respectively.

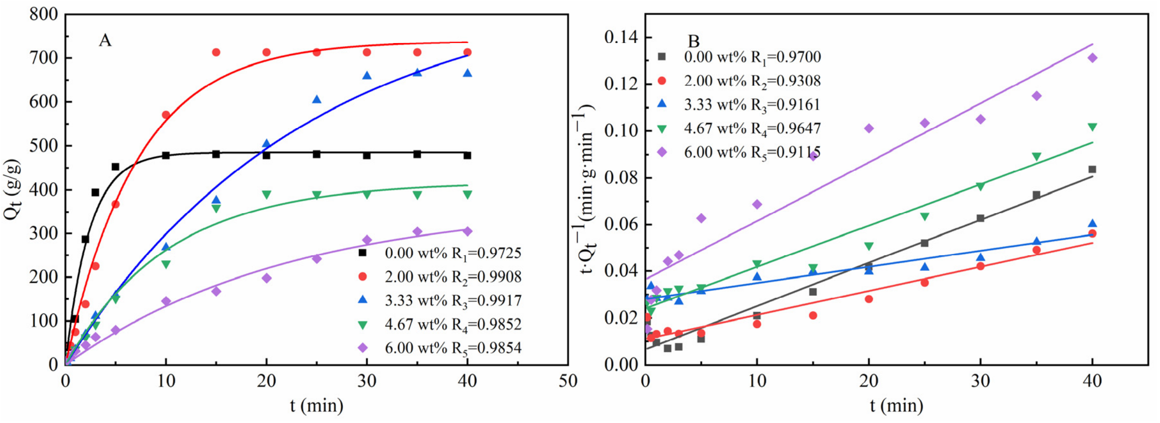

Figure S1. Fitting of CAAMC water absorption kinetic model in deionized water: pseudo-first-order kinetic model (A) and pseudo-second-order kinetic model (B).

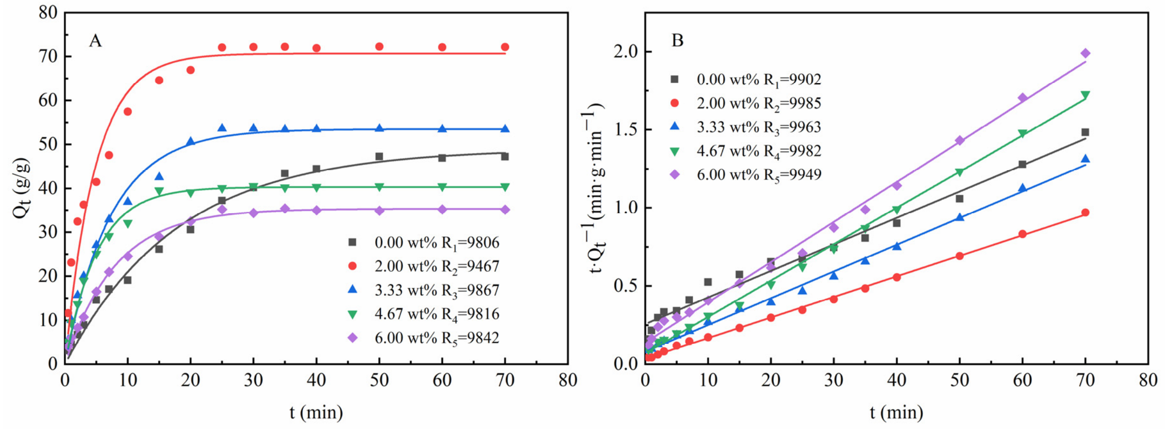

Figure S2. Fitting of water absorption kinetic model of CAAMC in 0.9 wt% NaCl solution: pseudo-first-order kinetic model (A) and pseudo-second-order kinetic model (B).

It can be seen from Fig.S1 that the correlation coefficient R^2 obtained by the pseudo-first-order kinetic equation fitting of the water absorption process of CAAMC in deionized water were greater than the correlation coefficient R^2 obtained by the pseudo-second-order kinetic equation fitting; therefore, the pseudo-first-order kinetic equation can better fit the water absorption process of CAAMC in deionized water. Additionally, it can be seen from Fig.S2 that the correlation coefficient R^2 obtained by the pseudo-first-order kinetic equation fitting of the water absorption process of CAAMC in 0.9 wt% NaCl solution were lower than the correlation coefficient obtained by the pseudo-second-order kinetic equation fitting R^2; therefore, the pseudo-second-order kinetic equation can better fit the water absorption process of CAAMC in 0.9 wt% NaCl solution. From the fitting results, it can be seen that CAAMC has different water absorption processes in deionized water and 0.9 wt% NaCl solution. This is mainly because, in the salt solution, the water absorption rate of CAAMC is not only affected by the volume swelling rate but also by salt ions, which reduce the adsorption capacity of the functional groups contained in CAAMC to water molecules.

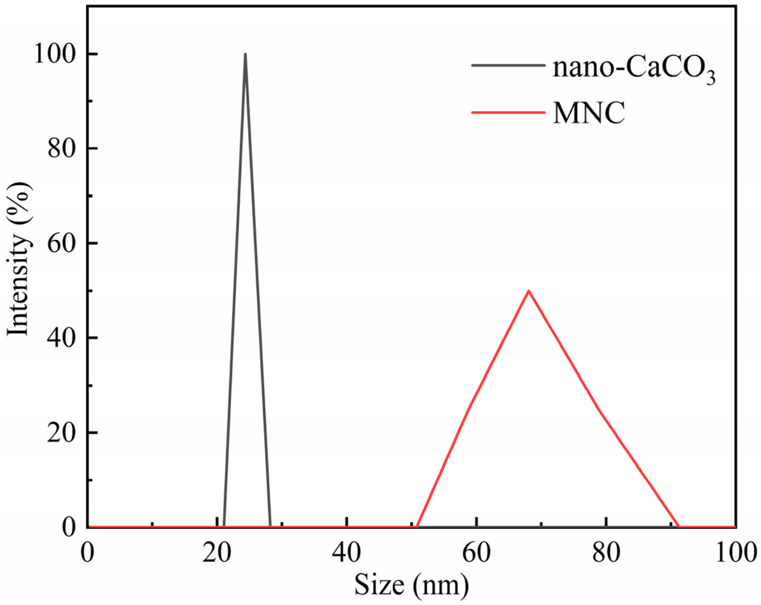

Figure S3. Particle size distribution of unmodified nano-$CaCO_3$ and MNC.

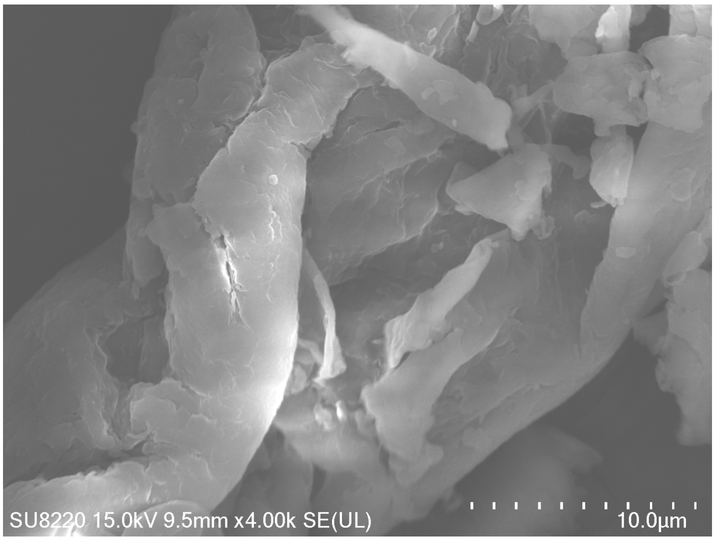

Figure S4. SEM images of the BC.

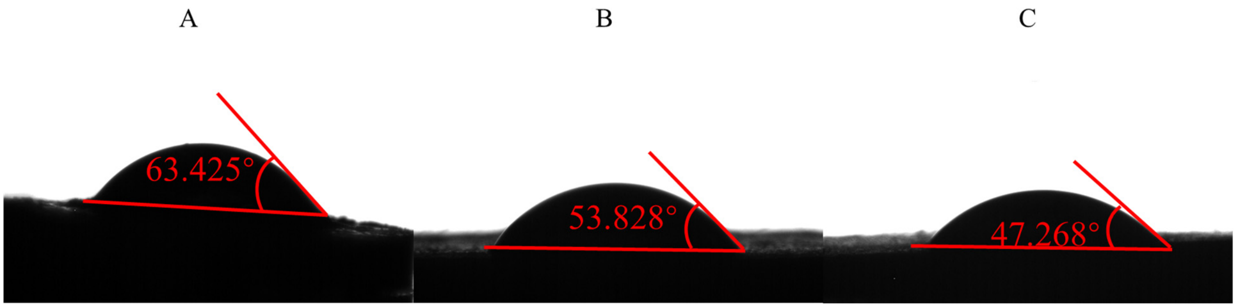

Figure S5. Photos of a water drop on the surface of bagasse (A), BC (B), and CAAMC (C) at the time of placing.

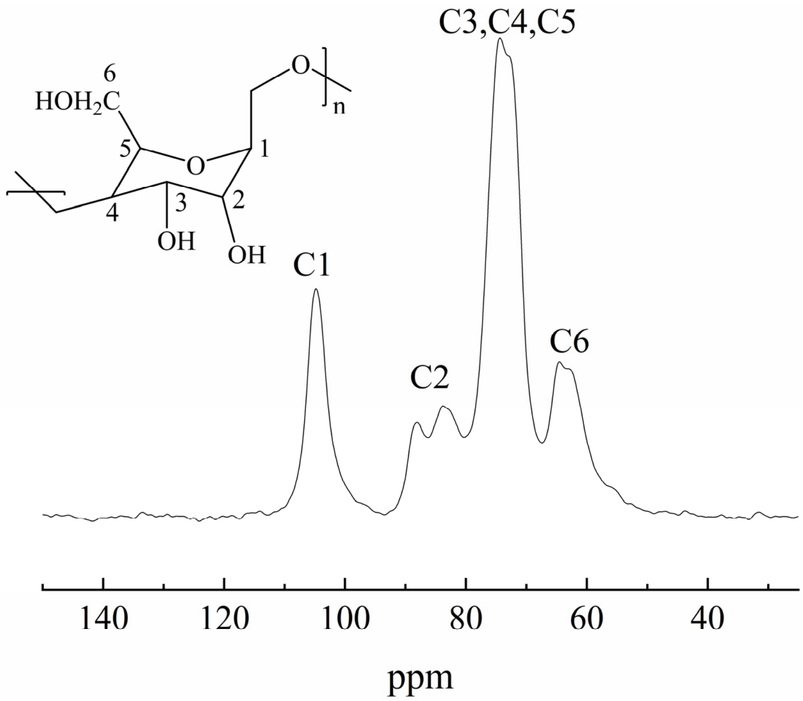

Figure S6. Solid-state ^{13}C NMR of BC.

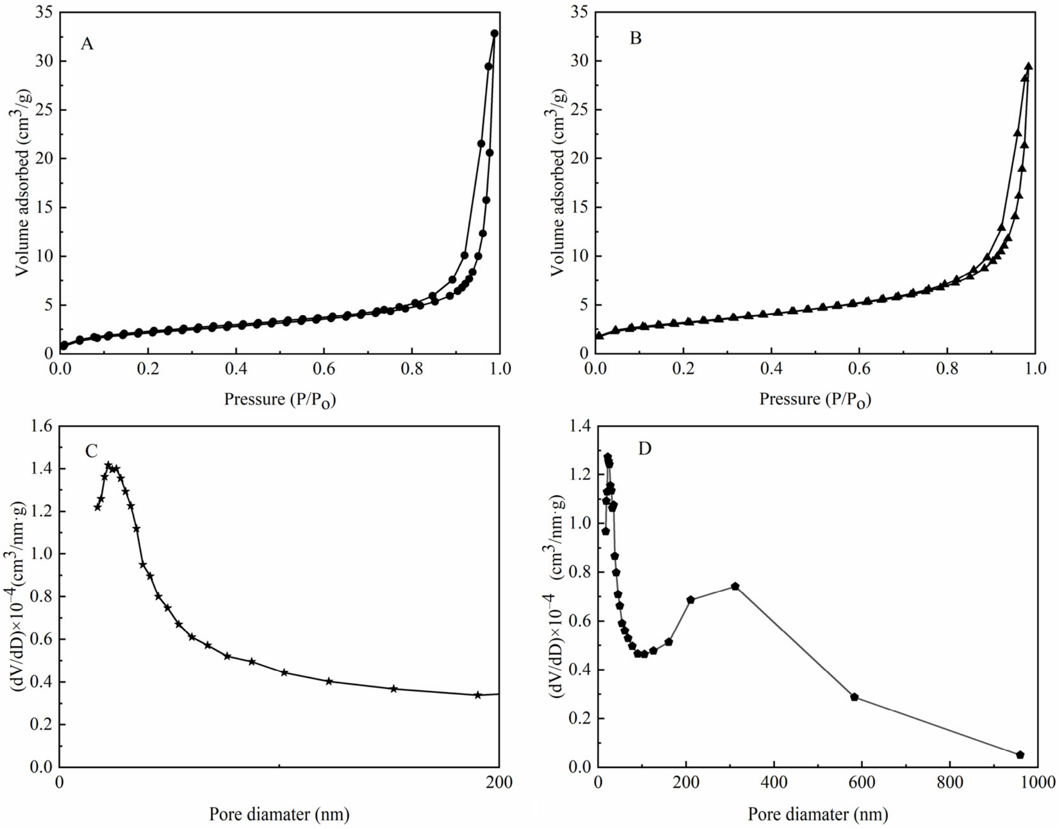

Figure S7. N_2 adsorption isotherms (nano-$CaCO_3$ (A) and MNC (B)) and pore size distributions (nano-$CaCO_3$ (C) and MNC (D)).

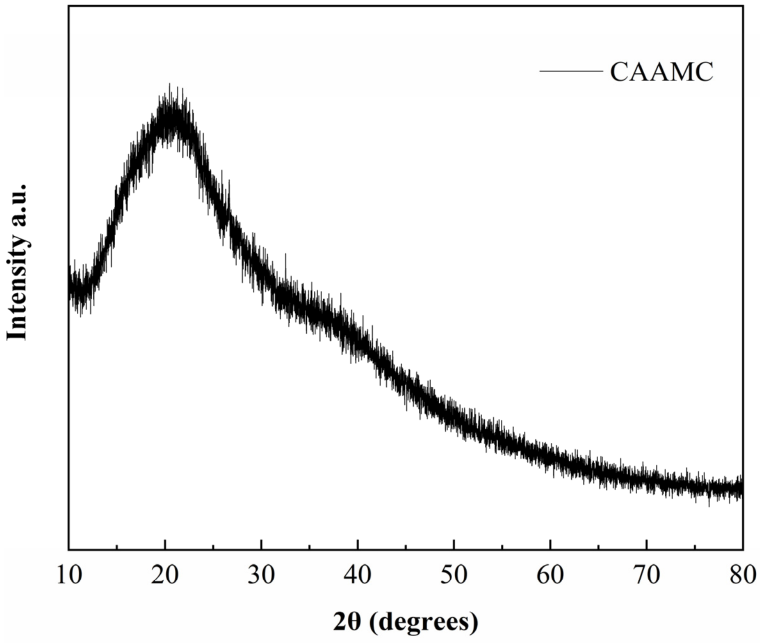

Figure S8. XRD pattern of CAAMC.
